# Thermal properties of thin films made from MoS_2_ nanoflakes and probed via statistical optothermal Raman method

**DOI:** 10.1038/s41598-019-49980-7

**Published:** 2019-09-16

**Authors:** Arkadiusz P. Gertych, Anna Łapińska, Karolina Czerniak-Łosiewicz, Anna Dużyńska, Mariusz Zdrojek, Jarosław Judek

**Affiliations:** Faculty of Physics, Warsaw University of Technology, Koszykowa 75, 00-662 Warsaw, Poland

**Keywords:** Applied physics, Two-dimensional materials, Characterization and analytical techniques

## Abstract

A deep understanding of the thermal properties of 2D materials is crucial to their implementation in electronic and optoelectronic devices. In this study, we investigated the macroscopic in-plane thermal conductivity (κ) and thermal interface conductance (g) of large-area (mm^2^) thin film made from MoS_2_ nanoflakes via liquid exfoliation and deposited on Si/SiO_2_ substrate. We found κ and g to be 1.5 W/mK and 0.23 MW/m^2^K, respectively. These values are much lower than those of single flakes. This difference shows the effects of interconnections between individual flakes on macroscopic thin film parameters. The properties of a Gaussian laser beam and statistical optothermal Raman mapping were used to obtain sample parameters and significantly improve measurement accuracy. This work demonstrates how to address crucial stability issues in light-sensitive materials and can be used to understand heat management in MoS_2_ and other 2D flake-based thin films.

## Introduction

Molybdenum disulphide is one of the most well-known members of the transition metal dichalcogenide (TMDC) family^1^. Mechanical exfoliation was applied to the layered structure of this material in order to isolate the first single-layer two dimensional (2D) semiconductor^2^. Over the years, various MoS_2_ properties have been exploited for a wide range of applications from electronic and optoelectronic devices such as transistors^3,4^, photodetectors^5,6^, and even integrated circuits^7^ to hydrogen production systems^8^ and lithium batteries^9^. Depending on the intended application, one can choose from several 2D molybdenum disulphide production methods^2,10–12^. Furthermore, MoS_2_ flakes can be fused to produce structures that are more complex, but also more useful than the original material, such as thin films. However, the resulting thermal and other physical properties can be significantly different from those of individual MoS_2_ crystals. The morphological structure and interactions between flakes can play a substantial role in the macroscopic parameters of such films. Thus, it is important to know how the applicable physical properties change upon moving from single flakes to thin films made from flakes.

Thermal conductivity (κ) and thermal interface conductance (g) are key thermal parameters and are essential to the efficient design and operation of many devices. These properties have been studied previously, but mostly with regard to MoS_2_ single crystals. Studies have been conducted on CVD and exfoliated mono- and multi-layer in-plane κ^13–20^ and g^14,15,19–24^ values. In- and out-of-plane bulk MoS_2_ κ anisotropy was measured^25,26^. Recent studies describe the dependence of the in-plane κ and g of mechanically exfoliated MoS_2_ on thickness^19^. The influence of defects on the in-plane κ of CVD monolayer MoS_2_ was also measured^27^. In addition, the in-plane thermal conductivity of a few-nm polycrystalline thin film created via the conversion method was studied as a function of grain size and orientation^11,28,29^. However, studies of the thermal properties of thin films of liquid-exfoliated and restacked MoS_2_ flakes are not currently available.

In this paper, we fill the gap identified above. We examine the thermal properties of MoS_2_ thin films produced via liquid exfoliation and vacuum filtration^12^. A state-of-the-art optothermal Raman technique was used to perform contactless, nondestructive characterization and simultaneous determination of κ and g^20,30,31^. We use statistical approach and spatially resolved Raman mapping to consider sample inhomogeneity and improve our measurement accuracy. The in-plane thermal conductivity and interfacial thermal conductance were measured to be 1.5 (9) W/mK and 0.23 (1) MW/m^2^K, respectively, at room temperature. We also show how uncertainty in the measured parameters affects the final outcome. These results help one to understand potential MoS_2_ thin film applications, contribute to better understanding of heat management, and can aid in measuring the thermal properties of other 2D flake-based materials.

## Theory

**Figure 1:**
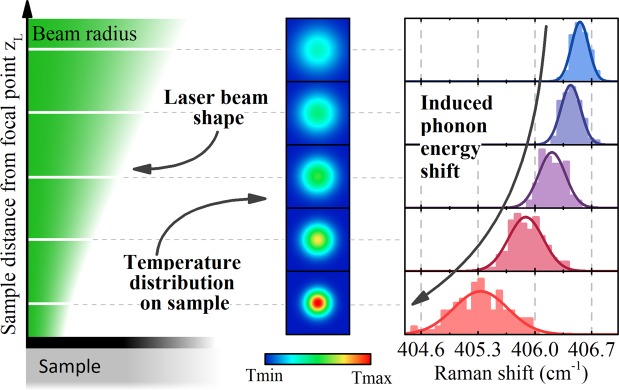
Concept of the experiment. Moving the sample from focal point of the laser beam causes the power and temperature distributions in the sample to change. The temperature increase induces a phonon energy shift, which can be observed using a Raman spectrometer.

In the steady state optothermal Raman method, a laser beam is used to both heat the material and excite phonons, whose energy can be correlated with temperature change in the sample (Fig. 1). The temperature increase distribution upon laser irradiation T(r) of a thin film deposited on a Si/SiO_2_ substrate depends on several parameters. These parameters can be divided into two groups. The first group includes the parameters of the thin film itself, such as κ, absorption (α), thickness (h), and g. The second includes parameters not related to the thin film, such as substrate thickness (h_SiO2_), substrate thermal conductivity (κ_SiO2_), incident laser power (P_L_), beam radius (r_0_), and laser power distribution. To calculate the temperature increase distribution in the thin layer, we can describe our system using thin film (Eq. (1)) and substrate (Eq. (2)) diffusive heat dissipation equations in the cylindrical coordinate system^20^:

1 $$\kappa h\frac{1}{r}\frac{\partial }{\partial r}(r\frac{\partial T(r)}{\partial r})-g(T(r)-{T}_{Si{O}_{2}}(r,0))+\frac{\alpha {P}_{L}}{{\rm{\pi }}{r}_{0}^{2}}\times {e}^{\frac{-{r}^{2}}{{r}_{0}^{2}}}=0$$

2 $${\kappa }_{Si{O}_{2}}\frac{1}{r}\frac{\partial }{\partial r}(r\frac{\partial {T}_{Si{O}_{2}}(r,z)}{\partial r})+{\kappa }_{Si{O}_{2}}\frac{{\partial }^{2}{T}_{Si{O}_{2}}(r,z)}{\partial {z}^{2}}=0$$

with the following boundary conditions:

3 $${{\kappa }_{Si{O}_{2}}\frac{\partial {T}_{Si{O}_{2}}(r,z)}{\partial z}|}_{z=0}-g(T(r)-{T}_{Si{O}_{2}}(r,0))=0$$

4 $${{T}_{Si{O}_{2}}(r,z)|}_{z=Si/Si{O}_{2}}=0$$

Examples of temperature increase distributions upon laser irradiation T(r) are presented in Supplementary Fig S1. We consider only in-plane thermal conductivity in thin film. This decision was justified by our simulation results. In our case of semi-transparent thin films, heat is evenly distributed throughout the layer thickness regardless of the cross-plane thermal conductivity. The assumption from Eq. (4), in which silicon is treated as an ideal heat sink, was justified by an experiment in which we did not observe an increase in the silicon layer temperature.

Measured via Raman spectroscopy, the temperature increase in thin film T_m_ is the weighted average of the real temperature spatial distribution T(r). We can calculate this as follows:

5 $${T}_{m}=\frac{{\int }_{0}^{\infty }rT(r){e}^{-{r}^{2}/{r}_{0}^{2}}dr}{{\int }_{0}^{\infty }r{e}^{-{r}^{2}/{r}_{0}^{2}}dr}$$

If all other system parameters are known, one can determine κ and g from Eqs (1–4) by measuring temperature increases for at least two different r_0_ values. The beam size is often altered by using different objectives with various numerical apertures (NA), working only in the focal plane^11,14,15,28,30,31^. Unfortunately, this approach is limited by the number of available objectives, which makes providing proper experimental conditions difficult. A simple but rarely used solution to this problem is to apply the properties of a Gaussian laser beam^20^. Using the Gaussian laser beam approximation, one can easily improve the beam radius control by relating the distance from the focal point z_L_ to r_0_:

6 $${r}_{0}=\sqrt{{r}_{m}^{2}+{({z}_{L}NA)}^{2}}$$

where r_m_ is the minimum beam radius at the focal point and NA is the numerical aperture of the objective. For *z*_*L*_ ≫ *r*_0_, which is the case in this work, the relationship takes the form:

7 $${r}_{0}\cong {z}_{L}NA$$

## Experimental

**Figure 2:**
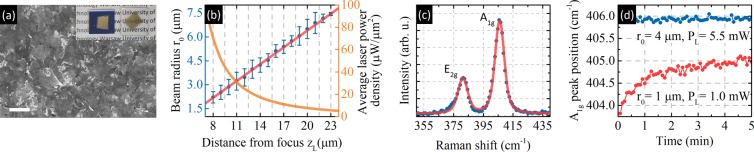
(**a**) SEM image of the sample. The scale bar is equal to 500 nm. Inset: photographs of MoS_2_ thin films on Si/SiO_2_ and glass. (**b**) Dependence of the beam radius (blue) and average laser power density (orange) on the distance to the focal point. Beam radii associated with different distances were measured via the knife-edge technique. The fit (red line) follows the Gaussian beam radius dependence from Eq. (7). (**c**) Sample Raman spectra with two Lorentz curves and a linear background. (**d**) Examples of stability tests performed to estimate the laser-induced damage threshold. Blue (red) points correspond to stable (unstable) measurement conditions.

MoS_2_ thin films were produced via liquid exfoliation and vacuum filtration using commercially available monolayer MoS_2_ powder (ACS Material, monolayer ratio >=90%, thickness ~1 nm, typical flake diameter: 1 μm–3 μm). A solution of powder in isopropanol was prepared via sonication and filtered through a cellulose membrane. The membrane was dissolved in acetone and the resulting thin film was cut and transferred to a substrates made of glass and Si/SiO_2_. Figure 2(a) shows an image from scanning electron microscope (SEM) used to characterize the film topography and demonstrate the random arrangement of flakes within the film. The inset shows photographs of samples produced via the same filtration process.

The thin film thickness was measured to be 65 nm via NT-MDT atomic force microscope (AFM). We prepared a patterned sample to improve our measurement accuracy. A 2 µm strip grid was created in our thin film using e-beam lithography and a SF_6_ plasma. Next, AFM measurements were performed over a 30 µm × 30 µm area to determine the average thin film height.

The optical properties of thin films on glass were analyzed using a photovoltaic response analyzer equipped with an integration sphere (PVE300 Bentham). A light beam (1 mm^2^) was used to obtain average thin film transmission, reflection, and absorption information. After accounting for reflection from the Si/SiO_2_ substrate, the total thin film absorption *α* was measured to be 51% of incident light at 514.5 nm.

Raman measurements were performed using a Renishaw inVia Raman spectrometer with a motorized XYZ stage that offered 100 nm resolution. All spectra were collected using a 50x objective and 514.5 nm laser excitation line in a backscattering configuration. The MoS_2_ (E_2g_, A_1g_) and Si bands were measured in one spectral window using a 3000 line/mm grating. We used circularly polarized light to avoid symmetry-based phenomena. The laser power was measured using an Ophir Nova II system with a PD-300 photodiode sensor. Sample temperature control was achieved using a Linkam DSC600 optical cell system with a temperature resolution of 0.1 K. Just before measurement, samples were annealed at 460 K for 1 h in air. The temperature in this step was set to ensure stability of our sample during experiment by avoiding transition effects like moisture evaporation and not cause considerable oxidation of the sample^32,33^. The beam radius was measured using a method similar to the knife edge technique^34^, in which the Raman scattering intensity of the Si band was measured across a sharp edge of gold thin film.

The nondestructive nature of the measurements was confirmed via stability tests with various combinations of beam radius and laser power. These tests consisted of 60 consecutive 5 s Raman measurements in the same location. Two Lorentz curves with linear background were fit to every spectrum. We extracted E_2g_ and A_1g_ peak positions, widths, and intensities as areas under curves. We considered the measurement conditions to be stable if the extracted parameters did not exhibit trends with respect to time.

In determining the thermal properties of MoS_2_ thin films, we focused on the A_1g_ peak because it was more intense and symmetric than the E_2g_ peak. We probed an area of 30 μm^2^ using 121 equally distributed points. Next, we averaged the data and treated the associated standard deviations as uncertainty in our measurements. All measurements performed during optothermal experiments used the same area of the sample.

## Results and Discussion

The measured relationship between z_L_ and r_0_ can be seen in Fig. 2(b). We performed 10 measurements for each z_L_. Five measurements were performed in each of the x and y directions to account for measurement uncertainty and possible beam asymmetry. The NA of the 50x objective used was determined by fitting Eq. (7) to the data. The experimental result NA_exp_ = 0.498(2) is in perfect agreement with the data provided by the manufacturer (NA_prod_ = 0.5). We also found that the uncertainty of r_0_ selected by changing z_L_ does not exceed 50 nm. The most important factor in optothermal Raman is the average power density. To better visualize the influence of z_L_ on this parameter, we calculated it and placed it on the same chart. Average temperature increase as a function of beam radius can be found in Supplementary Fig. S2. Figure 2(c) shows a sample MoS_2_ spectrum with fitted curves, while Fig. 2(d) shows two stability measurements. The importance of sample damage control can be seen clearly in the influence of stable and unstable measurement conditions on the A_1g_ peak position versus time.

**Figure 3:**
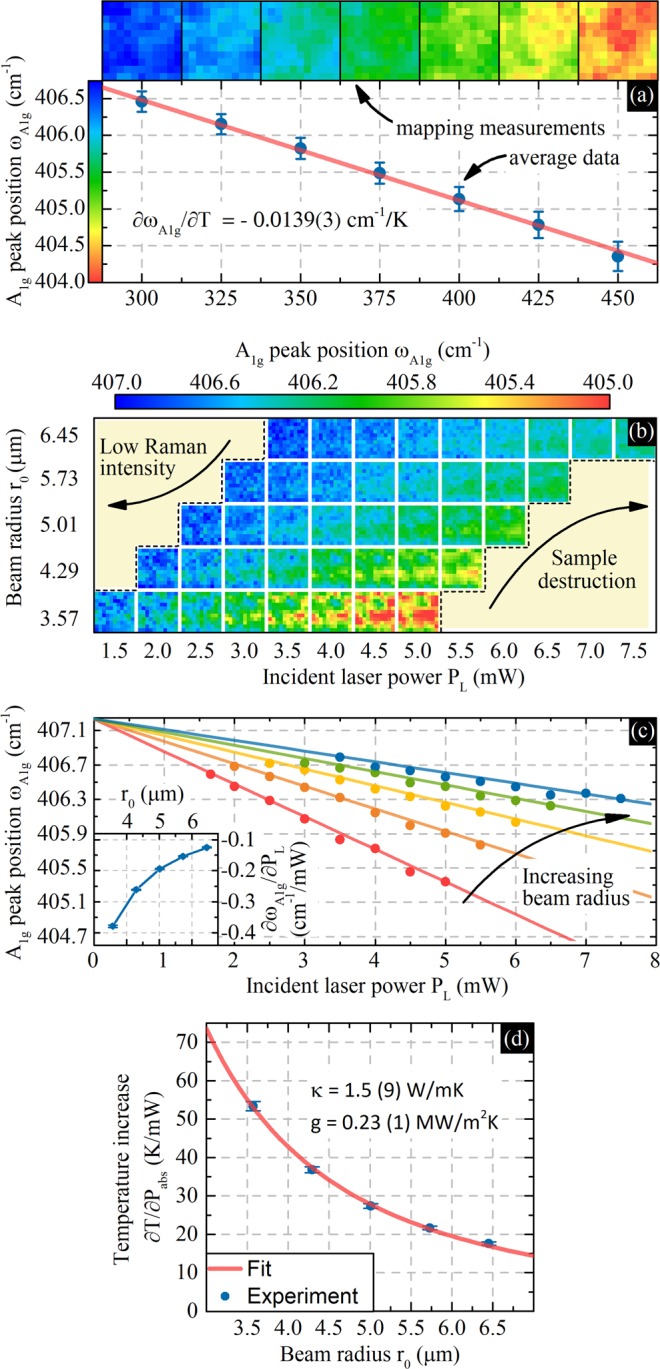
Optothermal Raman experiment. (**a**) Temperature dependence: mapping measurements and average data with linear fit. (**b**,**c**) Laser power and beam radius dependence: (**b**) mapping measurement and (**c**) average data with linear fits. To maintain figure clarity, uncertainty is not shown. The inset in (**c**) shows the derivative of the A_1g_ peak position with respect to the laser power for measured beam radii. (**d**) The derivative of temperature with respect to the absorbed laser power for five different beam radii with fitted theoretical curves.

Figure 3 shows data collected during optothermal experiments. The temperature dependence of the A_1g_ peak position (phonon energy) can be seen in Fig. 3(a). In this part of the experiment, we used a relatively large beam with a radius of about 5.5 μm and laser power of 5 mW to minimize laser heating of the sample. Spatial measurements are presented as heat maps. The scatter plot below shows average data with uncertainty and fit. The temperature dependence is well described by a linear function with a slope of χ_T_ = ∂ω_A1g_/∂T = −0.0139(3) cm^−1^/K.

Figure 3(b,c) show the dependence the A_1g_ peak position on the laser power density. We chose five beam sizes in order to control the power density. Each beam size was used with an appropriate laser power range. We selected measurement conditions using the following rules: 1. Each combination of laser power and beam size should cause a measurable temperature increase; 2. measurements should not induce sample damage; 3. Raman spectra collected in maps should allow one to probe the inhomogeneity of the thin film. Figure 3(b) shows the measured spatial distribution of the A_1g_ peak position as a function of selected parameters. The yellow areas correspond to conditions where reliable measurements were not possible due to low Raman intensity or sample destruction upon laser irradiation. Figure 3(c) shows calculated average Raman map data. We choose not to show uncertainty in order to maintain figure clarity. However, uncertainty will be discussed later in the text. For each beam radius, we use at least eight different laser power levels to reliably fit linear function. The fitting results can be seen as straight lines in the main figure, while the inset shows the power dependence χ_P_ = ∂ω_A1g_/∂P_L_ as a function of the beam radius. We note that all measurements were performed in the same area and thus in the zero laser power limit (P_L_ → 0), the A_1g_ peak position should converge to the same value for every beam radius. In our case this assumption is true within uncertainty limits, thus proving the non-destructive nature of the measurements and usefulness of the adopted methodology.

In addition, we want to draw attention to the statistical nature of the measurements and importance of proper sample damage control. Without this approach one would not be able to probe small peak position changes. For example, the induced phonon shift caused by modulating the incident laser power between 3.5 and 7.5 mW is only about 0.5 cm^−1^ for the largest beam size used in this experiment.

Figure 3(d) presents the derivative of the average temperature increase with respect to the laser power absorbed by the thin film for the five beam radii used in the experiment (For estimation of average temperature increase for every combination of beam radius and laser power see Supplementary Fig. S3). The temperature increase measured for each beam radius was calculated using data from Fig. 3(a–c) and the absorption coefficient. The following relation was used:

8 $${\frac{\partial T}{\partial {P}_{abs}}|}_{{r}_{0}=const}={\alpha }^{-1}\cdot {\chi }_{P}\cdot {\chi }_{T}^{-1}$$

A theoretical curve was obtained by solving Eqs (1–4) using the finite element method and adjusting the thermal conductivity and thermal interface conductance values to best fit the experimental data. Excellent agreement between experimental data and the theoretical curve is achieved for a κ of 1.5(9) W/mK and g of 0.23(1) MW/m^2^K.

As expected, the in-plane thermal conductivity of the MoS_2_ liquid-exfoliated thin film is at least an order of magnitude lower than those reported in the literature for crystalline monolayer, multilayer, or bulk MoS_2_ produced via mechanical exfoliation or CVD^13–20,25–27^. Similar results to our work were obtained by Sledzinska *et al*. using polycrystalline MoS_2_ thin films formed via conversion method^11,28^. They measured thermal conductivities from 2.0 (2) to 0.27 (15) W/mK that depended on the grain size and orientation. This suggests that the high thermal conductivities of the individual flakes in our sample are suppressed by the poor interconnections between them, as was the case with the polycrystalline film. The cross-plane thermal conductivity was reported as 0.28 W/mK^35^ in a study focused on the thermoelectric performance of thin films produced in a similar manner. This is considerably lower than the in-plane thermal conductivity measured in our experiment.

The literature contains few reports that consider the thermal interface conductance of MoS_2_ in various forms. Most reports focus on mono- or multi-layer MoS_2_ and provide values from 0.44 to 68.6 MW/m^2^K^14,15,19–24^. In this work, for thin film we obtained a smaller value of 0.23(1) MW/m^2^K. This might be due to a lower MoS_2_-substrate interface quality driven by smaller, more limited contact surface areas, the random orientations of individual flakes, and possible defects and impurities.

Next, we turn our attention to uncertainty and reliability of our measurements. First, we compare the standard deviations calculated from the mapping and stability tests conducted under the same measurement conditions. In all cases, the standard deviation calculated from the stability test is lower than that calculated from mapping measurements. This shows that the mapping data distribution comes not only from measurement uncertainty but also from inhomogeneity within the sample. Therefore, spatial measurements are needed to calculate average macroscopic parameters that consider sample inhomogeneity. This approach represents the best compromise for samples such as thin films made of 2D flakes where measuring thermal properties at every point is particularly difficult because of the need to know the absorption and thickness at every location.

**Figure 4:**
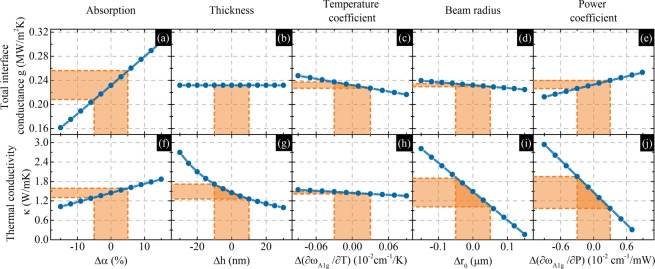
Influences of measured parameters on the thermal interface conductance (**a**–**e**) and thermal conductivity (**f**–**j**). On each x axis, zero corresponds to the measured value. Orange areas represent the uncertainties of parameters measured in the experiment.

We studied the sensitivity of our model to changes in the following experimentally measured parameters: absorption (α), thickness (h), phonon temperature dependence (χ_T_), beam size (r_0_) and phonon power dependence (χ_P_). The influences of these parameters on the thermal conductivity and thermal interface conductance are shown in Fig. 4(a–j). The orange areas correspond to the uncertainties in the measured parameters and how these uncertainties relate to κ and g. In the cases of r_0_ and χ_P_, we chose values with the highest uncertainties and sweep all r_0_ and χ_P_ values at once which corresponds to shifting all data points in Fig. 4(d) left-right or up-down. For clarity, we present the results in Table 1. We calculated the relative uncertainties of various parameters and compare their influences. We also calculated how changing each parameter by 1% of its relative uncertainty influences κ and g. The largest impacts on the final results are from r_0_ and χ_P_ for thermal conductivity and α for thermal interface conductance. Such analysis helps to design future experiments and shows the importance of a statistical approach that minimizes uncertainty in the most influential parameters.

**Table 1:** Uncertainty in measured parameters.

Parameter	Value	Uncertainty	Relative uncertainty (%)	Influence of 1% of relative uncertainty on g (MW/m^2^K)	Influence of 1% of relative uncertainty on κ (W/mK)
Absorption α	51%	5%	9.78	0.0025	0.01
Thickness h	65 nm	10 nm	15.38	0.0000	0.03
∂ω_A1g_/∂T	−0.0139 cm^−1^/K	0.00034 cm^−1^/K	2.45	0.0024	0.01
Beam radius r_0_	3.53 µm	0.05 µm	1.42	0.0017	0.31
∂ω_A1g_/∂P	−0.3789 cm^−1^/mW	0.0034 cm^−1^/mW	0.90	0.0086	0.62

We also estimated heat loss via radiation and convection in our experiment. We used a black body radiation model for the first and the procedure by Zhang *et al*. to consider convection from the material to the air for the second^15^. In each case, we based our calculations on the largest temperature difference between the sample and surroundings. We found that power dissipation via radiation and convection are at least three orders of magnitude smaller than phonon transport-related heat dissipation and can be ignored without having a meaningful impact on the final result.

## Conclusions

In conclusion, we measured the in-plane thermal conductivity and interfacial thermal conductance of a thin film made from MoS_2_ nanoflakes for the first time. An optothermal Raman technique was combined with mapping and statistical measurements to probe macroscopic sample parameters and increase accuracy. The keys to obtaining reliable results were proper assessment of sample damage and detailed uncertainty analysis. This work can be used as a reference for the design of future experiments that involve similar studies of 2D flake-based thin films or the influence of flake-to-flake interactions on macroscopic sample characteristics. It can also be a starting point for study of anisotropy of thermal conductivity in 2D flake based thin films through thickness dependent measurements.

## Supplementary information

Supplementary information

## Data Availability

The datasets generated and/or analysed during the current study are available from the corresponding author on reasonable request.
