# Supplementary material for: Thermal properties of thin films made from MoS2 nanoflakes and probed via statistical optothermal Raman method
Source: Sci Rep. 2019 Sep 16;9:13338. doi: 10.1038/s41598-019-49980-7 (PMC6746815; doi:10.1038/s41598-019-49980-7)
Supplement: Supplementary file 1 — Supplementary information [file 41598_2019_49980_MOESM1_ESM.pdf]

Supplementary information:

Thermal properties of thin films made from MoS_2 nanoflakes and probed via statistical optothermal Raman method

Arkadiusz P. Gertych*, Anna Łapińska, Karolina Czerniak-Łosiewicz, Anna Dużyńska, Mariusz Zdrojek, and Jarosław Judek

Faculty of Physics, Warsaw University of Technology, Koszykowa 75, 00-662 Warsaw, Poland
*Corresponding author: e- mail: arkadiusz.gertych@pw.edu.pl

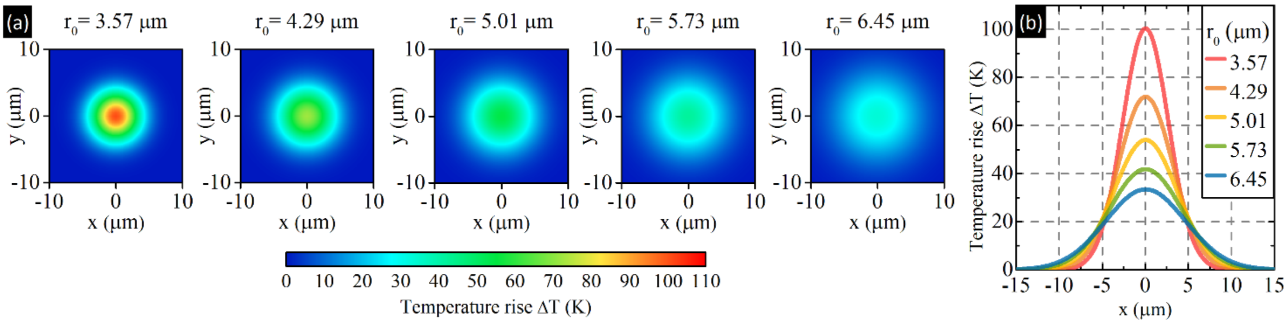

Supplementary Figure S1. Temperature increase distribution in MoS_2 thin film upon laser irradiation for five beam radii used in the experiments (Distance from focus z_L between 12 μm and 20 μm in equal steps). Results were obtained by solving heat dissipation equations with parameters measured in main article (κ = 1.5 W/mK, g = 0.23 MW/m^{2}K, thickness = 65 nm, 1 mW of absorbed laser power). (a) Spatial distribution of temperature increase. (b) X-profile of temperature increase.

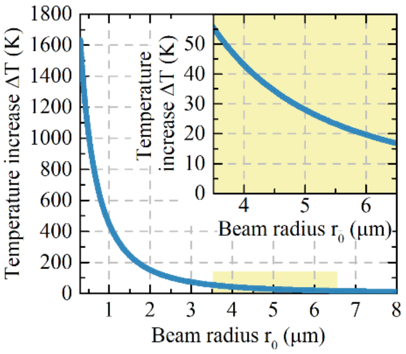

Supplementary Figure S2. Average temperature increase value estimation upon laser irradiation as a function of beam radius for thin film with parameters measured in main article (κ = 1.5 W/mK, g = 0.23 MW/m^{2}K, thickness = 65 nm, 1 mW of absorbed laser power). Yellow area and inset show average temperature increase for beam radius range used in the experiment.

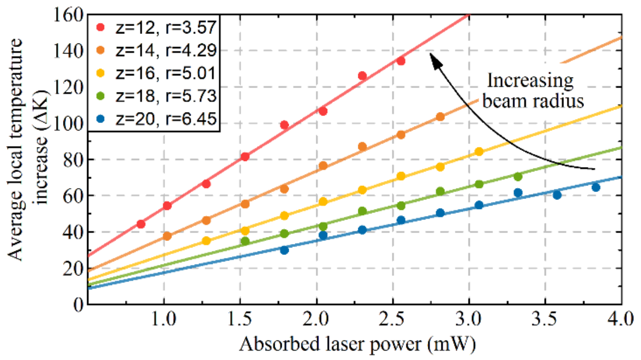

Supplementary Figure S3. Average local temperature increase estimated for every combination of beam radii and absorbed laser power used in the experiment. Distances from focus (z) and beam radii (r) in legend are in µm. Solid lines represent linear fits with slopes used in main article text to calculate thermal properties of the thin film (Fig 3d in main article).
